# Prevalence of Depression Amongst Adult Hemophilia Patients Registered With Hemophilia Foundation of Zambia

**DOI:** 10.1192/bjo.2024.134

**Published:** 2024-08-01

**Authors:** Dhruv Darji

**Affiliations:** University of Oxford, Oxford, United Kingdom. University of Lusaka, Lusaka, Zambia

## Abstract

**Aims:**

Main Objective

To assess the prevalence of depression (major depression) amongst adult hemophilia patients in Zambia.

Specific Objectives

1. To assess the prevalence of depression amongst adult hemophilia patients in Zambia using the Patient Health Questionnaire 9 (PHQ9) tool to screen for/diagnose depression.
2. To determine risk factors, amongst hemophilia patients, to developing depression.
3. To ascertain factors that significantly associate with depression amongst adult patients with hemophilia in Zambia.

**Methods:**

This was a quantitative cross-sectional study, conducted by administering the study questionnaire to collect data on demographic characteristics, clinical characteristics and the Patient Health Questionnaire – 9. A total of 59 adult patients with Hemophilia in Zambia, registered under the Hemophilia Foundation of Zambia were interviewed through the questionnaire. The data were analyzed using STATA 14. Descriptive analyses were done on the data, responses on PHQ-9 were totaled to assess for the prevalence of depression. Depression was defined as PHQ-9 ≥ 5; Major Depression as PHQ-9 ≥ 10. Pearson Chi-2 test was done to assess for associations and a logistic regression model was created to show the relationship between significant risk factors (independent variables) and depression.

**Results:**

59 participants were interviewed in this study. They were all male with an average age of 24.77 years from various parts of Zambia. 91.53% of the patients reported to have Hemophilia A, while 8.47% had Hemophilia B, there were no patients with Hemophilia C. The average PHQ-9 score was 8.66. 83.04% of participants had depressive symptoms (PHQ > 5); 44.06% having major depression and only 16.96% of the participants reported no depression. Number of painful bleeding episodes (OR = 2.063; P = 0.048) and difficulty in performing daily activity (OR = 4.311; P = 0.008) were significantly associated with a higher risk for major depression.

**Conclusion:**

There was a high prevalence of major depression (44.06%) amongst adult patients with hemophilia registered under the Hemophilia Foundation of Zambia. Hence there is need for addition of mental health care to the multidisciplinary management of adults with hemophilia for improved health outcomes due to the high prevalence of depression amongst this group. Additionally, patients who suffer many painful bleeding episodes must be prioritized candidates for mental health care.

